# On Filling Teeth

**Published:** 1847-10

**Authors:** E. Taylor

**Affiliations:** Cincinnati


### *On Filling Teeth, by* E. Taylor, *M. D. D. D. S., of Cincinnati.*

Messrs. Editors:—

As it is designed to make the Register a work of practical utility to the members of the profession, I propose to submit my views; and, as well as I can, my practice in the "nice and difficult operation" of filling teeth to the consideration of your readers.

I do not hold myself perfect in the art, nor capable of giving directions how to attain perfection in it, but by comparing modes of practice with others, and taking that which appears to be improvement in their practice, and blending the excellencies, I hope to attain to a good degree of perfection.

It is not in one single lump, but extracted from numerous lumps of ore, that the miner expects to fill his stores with treasures.—Even so it is in knowledge: it is "here a little, and there a little," that we pick up scraps which enrich mind. I only aim to deposite with you a few lumps of crude ore from which, if others can by smelting, refining and assaying, extract any of pure metal they are welcome of its use; and I hope others will cast in abundantly of purer ore, or may be of purer metal, and thus enrich your columns.

Well, sirs, the first thing toward filling a tooth is to have a patient, which by the bye is a matter of no inconsiderable importance. Instructions on this point are rather difficult to lay out. Probably, however, the best way to catch one would be to acquire a correct theoretrical and practical knowledge of the Dental profession; be provided with proper instruments and material; a comfortable room, and a suitable chair near where the animals are said to frequent. Tack up a "shingle" at your door, or have a decent doorplate; announce yourself to the public in a neat little card; keep closely to the office, and when "out" maintain a genteel deportment. This is rather an old formula, and its efficacy by many is much questioned; and the following recipe is said to be a certain specific. It, however, begins a step further back.

Take CARO VERDANT,<br>EFFRONS,<br>IGNAVUS,<br>AROGANTIA. } a. a.

Brayed well in a mortar until well mixed, and of proper consistence; mould to suit the fancy; rub a little, a very little will do, against a Dentist's shop until it *shines*, and you have the operator. Take a room at some second rate Hotel, or, as we have seen, have a small room fixed on wheels so as to be moveable. You have a better chance of "heading" them in this way, but sometimes the rapidity of steam might be found expedient—then it would be best to be unincumbered by such machinery. Have six or eight awls pointed up, one ounce of mercury, do. of silver or tin filings, a little mortar of some kind to mix it in, and a piece of buckskin to strain it through. Being thus *well* provided, announce to the world that the celebratea Monsieur —— has just arrived on the continent of North America with the most wonderful discovery that has marked this age of wonders, whereby, in justice to poor suffering humanity, he is in a moments time able infalliby and forever to cure all cases of Odontalgia (vulgarly called tooth ache), and save for life all teeth and stumps, by a painless operation, at one half the price usually charged by those calling themselves Dentists. That the numerous and pressing demands from the most distinguished men of other cities requiring his services, do not permit him to remain in this place to extend the benefits of his discovery but for a few days. All those therefore who wish to avoid the barbarous operation of extraction, and wishing to *save* their teeth, will call at once on Monsieur —— and examine his credentials, as he has certificates of the most astonishing cures from Physicians, Clergymen, Lawyers, Judges, Honorables, &c., suspended in frames around his room. Have your door and windows covered with signs, swagger about the bar-rooms, put on a foreign air, puff Havanas, and—but probably this will suffice. One great objection to this course is that it brings in a set of customers whose ears are sometimes inconveniently long; but they will generally suit the operator very well.

The patient being had, the next thing is to have him or her, as the case may be, well seated. For this purpose every Dentist should have a suitable chair, so as to render the patient as comfortable as may be, and yet have the head placed in a condition favorable for the operation, and steady and secure. There are several qualities desirable in an operating chair. The seat should be moveable, or a supply of cushions kept so as to raise or lower, as may be desired to bring the heads of the different patients to about the same height. We soon become accustomed to operating at a certain height, and will soon discover that we can operate at this height with more ease and greater facility than at any other. The height that I prefer is, to have the mouth on about a parallel with the middle of the humerus, when standing straight. At this height I have but little stooping, and yet it is not so high as to weary the arm in operating. Some situations on the lower teeth will require the patient to be seated lower, or a platform will be needed to stand on. We sometimes wish to throw the patient further back; the chair should therefore have this facility, as also to throw the head back or forward by a moveable head piece. It should be strong and set firmly.

To have a good light is also a very important matter—not so effulgent as to dazzle, but strong and steady. The eye of the operator is probably more tried than any other member of the body, and its comfort should be much consulted. I have operated by the light from every point, but decidedly prefer an unobstructed northern as sufficiently strong and far more comfortable to the eye than any other.

A suitable spittoon should also be provided, something that will be neat and convenient; and what is also very important, particularly in warm weather, is that it be kept clean and sweet. Indeed the whole arrangement of the room should be to make it in appearance and reality comfortable and *nice.*

There must also be a supply of proper instruments--some of these I purpose describing more particularly hereafter. They should be conveniently placed, and so arranged that the instrument desired may be picked up at once. "A place for every thing, and every thing in its place," should not only be the motto but the practice of every Dentist. I deem it the best, and it is certainly most convenient to have the instruments so placed that they may be picked up without leaving the side, or being obliged to withdraw the other hand from the mouth of the patient. The case which I at present use, I think the most convenient of any that I have seen. It is in height about to my elbow, some twenty inches wide, and somewhat over four feet in length; and is in external appearance something like a plain sideboard. The top is a lid hinged on, and when open is held by a stop-hinge, which exposes all my excavators, pluggers, scalers, mirrors, &c., cased as convenience demands. Below this, I have a double range of drawers, six in all, in which I keep my forceps, foil, points, napkins, cotton, pivot teeth, and such instruments as I do not care to case. Below are two doors and shelves for such uses as may be desired. I have it placedat the side of my chair, and stand between its front and the patient, having it about thirty inches from the chair. It is so high that I do not have to stoop to pick up an instrument, and so close that I can reach any one in it without letting go the mouth of the patient. The drawers are secured by "drops" through their front ends, and the lid being down and locked, all are secure. Now we are ready for operating.

*(To be continued.)*

